# Hostility and negative expectations about the future in mental disorders

**DOI:** 10.1192/j.eurpsy.2025.2205

**Published:** 2025-08-26

**Authors:** T. I. Medvedeva, S. N. Enikolopov, O. Y. Vorontsova, O. M. Boyko

**Affiliations:** 1 Clinical psychology, Mental Health Research Center, Moscow, Russian Federation

## Abstract

**Introduction:**

Studies shows a connection between hostility and the severity of psychopathological symptoms. When psychopathological symptoms (mainly depressive) are expressed, hostility towards one’s own “I”, other people, and generalized impersonal hostility in the form of a sense of injustice, ill will of the surrounding world, and a negative assessment of the subjective future are combined. the connection between hostility and a negative assessment of the future requires empirical confirmation.

**Objectives:**

The **aim** of the study was to analyze the connection between hostility and a negative assessment of the future.

**Methods:**

N=37 people hospitalized in the clinic of the *Mental Health Research Center* (16 men and 21 women) with a diagnosis of schizophrenia and affective spectrum. Methods: SCL-90R, BPAQ-24 (Buss, Perry), All the subjects wrote a short essay “Me, others, the world”, attitude to the future was assessed by a group of answers in the modified Sentence Completion Test (Sacks, Levy). The subjects were divided into three subgroups: “positive expectations of the future” (N=16, mean age 24.87±8.20), “neutral future” (N=10, mean age 21.89±8.08), “negative assessment of the future” (N=11, mean age 21.45±4.82). The presence of a trend in changing parameters depending on the attitude to the future - Jonckheere-Terpstra Test, comparison of subgroups by parameters of qualitative assessment of the essay - Chi-Square Tests were used.

**Results:**

The analysis showed an increase in “hostility” (BPAQ-24) with a change in attitude to the future from negative to neutral and positive. With a negative attitude towards the future, there were the highest rates of “hostility” (24.82 ± 4.26, 17.60 ± 5.58, 16.40 ± 4.63, Std. J–Tstat. = -3.44, p = .001). It was revealed that negative expectations of the future are associated with the presence of problems in interpersonal relationships in the present, “Interpersonal Sensitivity” (SCL-90) is increased (10.82 ± 7.37, 7.00 ± 6.88, 4.87 ± 6.81, Std. J–Tstat. = -2.348, p = .019), individuals with high rates are distinguished by negative expectations regarding interpersonal interaction and any communications with other people. The analysis of the parameters of the qualitative analysis of the essays in the subgroups showed that only with a negative attitude towards the future there is a mention of the fragility and instability of the world (36%, p=.007), statistically more often mention the topic of suicide, death, “no place in this world” (80% compared to 16% and 22%, p=.049), the topic of “rejection” (p=.025), the frequency of expectation of a negative assessment of oneself by other people (, p=.004).

**Conclusions:**

Results confirm the hypothesis about the relationship between hostility and a negative attitude towards the future and allow to assume that a common factor for both hostility and a negative attitude towards the future in mental pathology are problematic interpersonal relationships.

**Disclosure of Interest:**

None Declared

